# Vascular development and hemodynamic force in the mouse yolk sac

**DOI:** 10.3389/fphys.2014.00308

**Published:** 2014-08-20

**Authors:** Monica D. Garcia, Irina V. Larina

**Affiliations:** Department of Molecular Physiology and Biophysics, Baylor College of MedicineHouston, TX, USA

**Keywords:** Yolk Sac, live imaging, vascular remodeling, mouse development, Hemodynamics

## Abstract

Vascular remodeling of the mouse embryonic yolk sac is a highly dynamic process dependent on multiple genetic signaling pathways as well as biomechanical factors regulating proliferation, differentiation, migration, cell-cell, and cell-matrix interactions. During this early developmental window, the initial primitive vascular network of the yolk sac undergoes a dynamic remodeling process concurrent with the onset of blood flow, in which endothelial cells establish a branched, hierarchical structure of large vessels and smaller capillary beds. In this review, we will describe the molecular and biomechanical regulators which guide vascular remodeling in the mouse embryonic yolk sac, as well as live imaging methods for characterizing endothelial cell and hemodynamic function in cultured embryos.

## Introduction

The first functional organ system to form in mammalian embryos is the cardiovascular system. The early establishment of the cardiovascular network during development is a complex, morphogenetic process, which requires the regulation of multiple cell types and the activation of several signal transduction pathways. If this network is not established properly, it affects organ development and embryo viability (Coultas et al., 2005; Udan et al., 2013a). Furthermore, maintenance and stabilization of this complex cardiovascular network must occur throughout adulthood, as impairments in vessel integrity and hemodynamic function can result in poor health or early death (Carmeliet, 2003).

Elucidating the physical and molecular cues required to trigger the morphogenetic events that occur during vascular development has been a significant challenge to developmental biologists, cancer biologists, physiologists, and scientists of many other disciplines for centuries, and has been speculated upon since the time of Aristotle (Risau, 1997). Of particular importance is understanding how the physical cues imparted by hemodynamic force affect the regulatory events and signaling pathways controlling embryonic vessel development. Defining these mechanisms is critical for determining how a normal vascular network develops and how aberrant vascular development can contribute to disease. Importantly, understanding the mechanisms by which a healthy vasculature is established will aid in the development of clinical and therapeutic interventions for the prevention and treatment of cardiovascular diseases.

## Vasculogenesis in the mouse embryo

**Figure 1:**
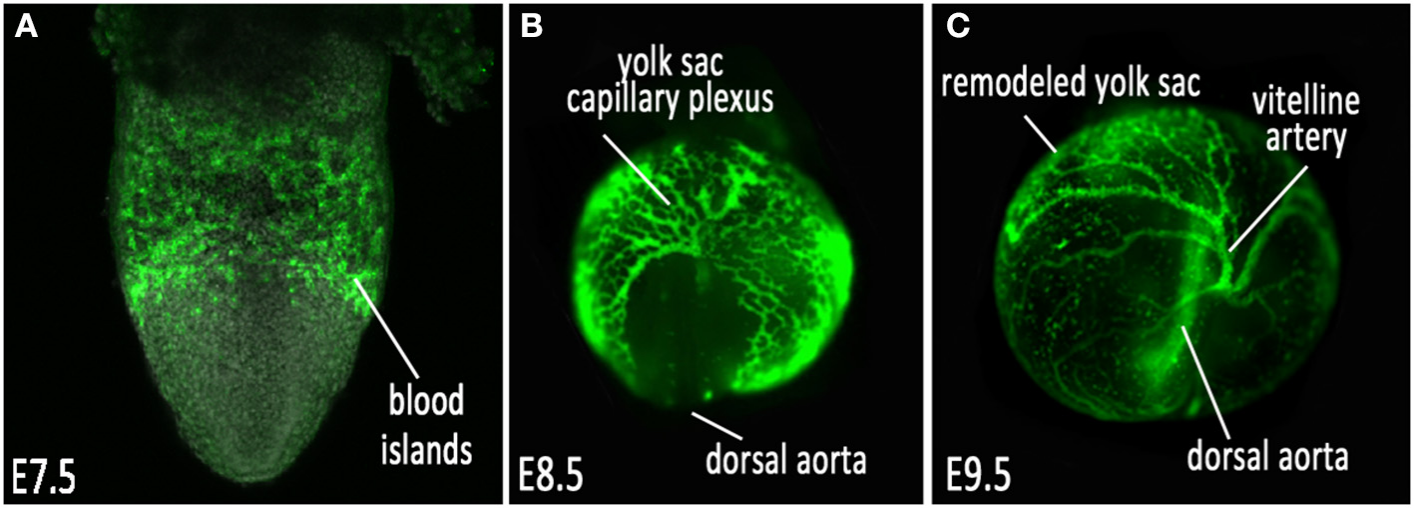
**Vascular development of the mouse embryonic yolk sac. (A)** VEGFR2/Flk1 expression labeling nascent vessels in the blood islands at E7.5; **(B)** ε-globin-GFP labeled erythroblasts filling the primitive capillary plexus at E8.5, and **(C)** after vascular remodeling at E9.5.

The mouse is a leading model system for studying the physical and molecular regulation of vascular development. The availability of genetic manipulation in mice has provided a wealth of information about the molecular pathways involved in the establishment, remodeling and maintenance of the cardiovascular network. In the mouse embryo, the initial establishment of blood vessels occurs in the absence of hemodynamic force. Cells that give rise to the first primitive vessels located in the yolk sac begin to form during gastrulation, which occurs on mouse embryonic day 6.5 (E6.5). During this time, the epiblast, which is a single epithelial cell layer, undergoes an epithelial-to-mesenchymal transition to form the primitive streak, which gives rise to the germ layers of the embryo proper—the mesoderm, definitive endoderm, and embryonic ectoderm (Nowotschin and Hadjantonakis, 2010; Solnica-Krezel and Sepich, 2012). Vasculogenesis, the *de novo* formation of a network of blood vessels, is the first phase of vessel development, where blood and endothelial cell precursors (angioblasts) migrate from the mesoderm, aggregate and form the blood islands located in the proximal extraembryonic yolk sac by E7.5 (Figure 1A) (Risau and Flamme, 1995; Shalaby et al., 1997). After the blood islands have formed, angioblasts located at the outer edges divide and differentiate into endothelial cells and migrate distally into the yolk sac, forming a simple capillary network that encompasses the yolk sac. This network of interconnected rudimentary small vessels, homogeneous in shape and size, is the primitive capillary plexus (Figure 1B) (Drake and Fleming, 2000; Coultas et al., 2005; Udan et al., 2013a).

The initial establishment of the capillary plexus during vasculogenesis is largely dependent on activation of the vascular endothelial growth factor (VEGF) signaling pathway. Several genetic studies in mice identified vascular endothelial receptor 2 (VEGFR2/Flk1) as a regulator of *de novo* vasculogenesis, critical for the development of both blood cells and the primitive capillary plexus during embryogenesis (Klagsbrun and Soker, 1993; Dvorak et al., 1995; Poole et al., 2001; Coultas et al., 2005). During the *de novo* establishment of the capillary plexus of the yolk sac, as well as the early development of the vasculature within the embryo proper, VEGF signaling is finely balanced to allow for the migration and proliferation of endothelial cells into the primitive vascular network (Leung et al., 1989; Klagsbrun and Soker, 1993; Dvorak et al., 1995; Petrova et al., 1999; Coultas et al., 2005; Shibuya and Claesson-Welsh, 2006). Furthermore, the Scl/T-cell acute lymphoblastic leukemia transcription factor (Scl), a member of the basic-helix-loop-helix (bHLH) transcription factor family, has been identified in the specification of Flk1 positive multipotent progenitors, which give rise to both blood and endothelial cells (Robb et al., 1995; Shivdasani et al., 1995; Porcher et al., 1996; Visvader et al., 1998). In fact, putative Scl binding sites have been found on the Flk1 promoter, suggesting that Scl directly acts as a regulator of Flk1 expression and multipotent properties of early endothelial and hematopoietic progenitor cells, critical for the establishment of the initial capillary plexus during vasculogenesis (Kappel et al., 2000). The development of this capillary network in the yolk sac is one of the first steps in the sequence of highly dynamic morphogenetic events that occur during early vascular development.

## Angiogenesis and vascular remodeling in the mouse yolk sac

Once the primitive capillary plexus of the yolk sac is established by about E8.5, this honeycomb-like network of vessels undergo rapid morphological changes to adjust to the demands of the expanding yolk sac and growing embryo. This is an angiogenic process, where the established vessels remodel to expand and increase in diameter, and smaller vessels fuse to form the large major vessels of the yolk sac and embryo (Udan et al., 2013a). Concurrently, other vessels undergo pruning to eliminate excessive branches, intussusception to create branch points in large vessels, and nascent vessels sprout from existing larger vessels to form small capillary beds. This morphogenetic process, known as vascular remodeling, occurs between E8.5 and E9.5, and results in a hierarchically branched, highly organized vascular network (Figure 1C) that allows vessels to become low-resistance conduits for the transport of blood, oxygen, nutrients and waste to and from the yolk sac and embryo proper. This remodeling process is a critical time point required for the ability of the cardiovascular system to function and support the growing embryo; if the vessels are not remodeled correctly, the viability of the embryo is no longer sustainable. This process is regulated by both molecular and biomechanical signaling.

Some of the most studied and earliest identified molecular signaling pathways that are essential for angiogenesis are the Tie family of receptor tyrosine kinases. Expression analysis of Tie1 and Tie2 receptors show localization within the vasculature from the onset of angioblast specification (E7.0), continuing on through development and into adulthood. (Dumont et al., 1992, 1995; Sato et al., 1993; Schnurch and Risau, 1993; Korhonen et al., 1994). Furthermore, targeted mutations of Tie receptors in mice revealed significant impairments on vascular development during angiogenic processes, including yolk sac vascular remodeling (Dumont et al., 1994; Sato et al., 1995). Although Tie receptors were initially thought to be orphan receptors, extensive studies have identified four ligands that can bind to Tie receptors: angiopoietins 1–4, (Davis et al., 1996; Suri et al., 1996; Maisonpierre et al., 1997; Valenzuela et al., 1999). These ligands have also been shown to be necessary for the regulation of yolk sac vascular remodeling in the mouse embryo (Davis et al., 1996; Maisonpierre et al., 1997; Gale et al., 2002). These data identify the Tie family of receptors and ligands as critical molecular regulators of vascular remodeling during angiogenesis, after the initial establishment of the capillary plexus during vasculogenesis.

Another example of the molecular regulation of yolk sac angiogenesis and vascular remodeling is the transforming growth factor β superfamily (TGFβ), which encompasses transforming growth factors and receptors, bone morphogenetic proteins and activins (Jakobsson and van Meeteren, 2013). Homozygous deletion of TGFβ family members results in embryonic lethality by E10.5–E11.5, at which point the mice are shown to have an unremodeled yolk sac vasculature, defects in endothelial and hematopoietic differentiation, delayed angiogenesis and weak vessels (Letterio et al., 1994; Dickson et al., 1995; Oshima et al., 1996; Yang et al., 1999; Oh et al., 2000; Urness et al., 2000; Larsson et al., 2001).

These genetic experiments provide examples of the individual contribution of members of the TGFβ pathway to remodeling of the vasculature of the yolk sac and embryo. The fine balance of activation, localization and balance of TGFβ ligands, receptors, and downstream signal transduction molecules have all been shown to be critical regulators of angiogenesis, vascular remodeling and vessel stability in the mouse embryo during development.

At the conclusion of vascular remodeling, the vasculature of the yolk sac and embryo are subdivided into a highly organized, hierarchical network of structurally and functionally distinct arteries and veins, each with its own specialized purpose for the delivery of oxygen and nutrients to the developing embryo. Although it has been hypothesized that the differential effects of hemodynamic force of blood flow on developing vessels is a major factor for the molecular distinction between arteries and veins (le Noble et al., 2004), molecular markers of arterial and venous cell fate have been identified in endothelial cells before the onset of hemodynamic force.

The earliest identified and most studied factors which regulate arterial/venous identity are the Eph receptors, a large family of receptor tyrosine kinases and their membrane-bound ligands, the ephrins (Klein, 2012). Analysis of *EphrinB2* and its receptor *EphB4* at the earliest stages of vascular development revealed compartmentalized expression of *EphrinB2* within the endothelial cells of developing arteries, whereas expression of *EphB4* is localized to venous endothelial cells (Wang et al., 1998), suggesting that the differences between arteries and veins are genetically pre-determined and are critical for proper vascular function during development. Furthermore, the phenotypic analysis of several knockout mice within the Eph family of receptors and ligands confirmed their essential role in vascular morphogenesis (Wang et al., 1998; Gerety et al., 1999; Adams et al., 2001; Gerety and Anderson, 2002). Researchers in the field describe the interaction between ephrinB2 and EphB4 as reciprocal, in which signaling between the arterial and venous compartments allows for the separation of arterial and venous cell fates, downstream of pathways that are necessary for the establishment of arterial and venous progenitors (Klein, 2012).

The Notch signaling pathway has been implicated as regulator of arterial and venous cell fate upstream of ephrin signaling. This family consists of the Notch receptors (Notch 1–4), which interact with the ligands Delta-like (Dll) 1, 3, 4, Jagged1 and Jagged2. Upon interaction between the ligand and its receptor, the intracellular domain of the receptor is cleaved, where it can translocate to the nucleus and activate transcription of target genes (Gridley, 2010). Analysis of Notch ligands and their receptors have identified expression in the endothelium, with many family members restricted to the arterial compartment (Villa et al., 2001). Targeted mutation of Notch pathway members in mice has provided further evidence that this pathway is key in regulating arterial endothelial cell identity and yolk sac vascular morphology (Conlon et al., 1995; Xue et al., 1999; Krebs et al., 2000; Fischer et al., 2004; Gale et al., 2004).

While the specification of arterial identity is regulated by a number of different transcription factors and signaling molecules, specification of venous cell fate is primarily mediated through the orphan nuclear receptor chicken ovalbumin upstream promoter-transcription factor II (CoupTFII). Targeted deletion of *CoupTFII* in mice results in an unremodeled yolk sac vasculature and defects in cardiac morphogenesis (Pereira et al., 1999). *CoupTFII* is expressed solely in the venous compartment of the endothelium, and upon conditional deletion of *CoupTFII* in endothelial cells, expression of arterial markers is observed within venous endothelial cells. Conversely, ectopic expression of *CoupTFII* results in the fusion of arteries and veins. The authors conclude that CoupTFII functions cell autonomously to suppress the activation of arterial regulators to maintain venous cell fate (You et al., 2005).

The specification and maintenance of arterial and venous identity is critical for viability as the embryo continues to grow. In the absence of functional arteries and veins, circulation can become impaired, highlighting the importance of the early genetic specification and patterning of these specialized endothelial cells before the onset of blood flow. Furthermore, after remodeling, the newly formed vessels of the yolk sac and embryo begin to stabilize through the recruitment of mural cells to the endothelium, including smooth muscle cells and perictyes. This recruitment is initiated by the secretion of platelet derived growth factor B (PDGFB) from endothelial cells, which in turn binds to PDGF receptors on the mural cell surface and allows them to contribute to the stabilization and formation of the vessel wall (Armulik et al., 2005; Gaengel et al., 2009).

## The regulation of endothelial cells by hemodynamic force during vascular remodeling

Physical forces imparted by blood flow are known to regulate vascular remodeling within the yolk sac and embryo proper (Lucitti et al., 2007; Culver and Dickinson, 2010). *In vitro* analysis of how endothelial cells respond to mechanical force has been insightful in identifying mechanosensitive signal transduction pathways that can modulate endothelial cell morphology and function (Tzima et al., 2001, 2002, 2003; Osawa et al., 2002; Newman and Newman, 2003; Lehoux et al., 2006). For example, the application of shear stress, the frictional force of flowing blood dragging along the vessel wall, to cultured endothelial cells has been shown to activate a signal transduction complex composed of three receptors, VEGFR2 (Flk1), vascular endothelial cell cadherin (VE-cadherin), and platelet endothelial cell adhesion molecule 1 (PECAM1) *in vitro* (Tzima et al., 2005). When a confluent monolayer of endothelial cells in culture is exposed to laminar (steady, non-turbulent) flow, endothelial cells realign parallel to the direction of flow. Although previous work had indicated this phenomenon was dependent solely on the activation of the phosphatidylinositol-3-OH kinase (PI3K) pathway through Flk1 (Shay-Salit et al., 2002), the authors demonstrated that upon deletion of either *VE-cadherin* or *PECAM1* in confluent endothelial monolayers exposed to flow, the cells were no longer able to respond to the shear stress and did not realign in the direction of flow. Furthermore, upon overexpression of *Flk1, PECAM1*, and *VE-cadherin* in cells which do not respond to mechanical shear stress normally, cells realigned parallel to the direction of flow, becoming mechanosensitive (Tzima et al., 2005). These data provided evidence of a molecular complex that is triggered solely by mechanical force, which serves as a necessary cue for the ability of endothelial cells to change morphology and polarity within a cultured monolayer. The authors concluded that the mechanism for activation of this mechanosensory complex is through direct transmission of mechanical force via PECAM1 to VE-cadherin, which acts as an adaptor protein activating Flk1 and catalyzing the activation of the PI3K signaling cascade. Furthermore, *in vitro* experiments using FRET (fluorescence resonance energy transfer) biosensors showed increased force acting on PECAM1 at junctions upon the addition of shear stress, further supporting the idea of PECAM1 as a mechanosensitive signaling factor (Conway et al., 2013). Whether this complex is activated in a similar way *in vivo* has yet to be determined, but it is known that some of these receptors are essential for embryonic vascular development. As described earlier, Flk1 is critical for the earliest stages of both vasculogenesis and hematopoiesis (Shalaby et al., 1997), and VE-cadherin is necessary for vascular morphogenesis (Gory-Faure et al., 1999). However, while PECAM1 is expressed in the endothelium during embryonic vascular development, genetic deletion of this gene shows no deficiencies in vasculogenesis, angiogenesis, or vascular remodeling (Duncan et al., 1999), indicating the mechanism by which hemodynamic force regulates vascular remodeling *in vivo* may rely on additional factors. Furthermore, several *in vitro* experiments performed to determine the mechanism behind the mechanosensitivity of endothelial cells have identified a wide array of proteins, including ion channels, G-protein coupled receptors, nitric oxide release molecules, and signaling transduction pathways, all of which may play a potential role in the regulation of vascular remodeling *in vivo* (Lehoux et al., 2006; Culver and Dickinson, 2010).

The characterization and quantification of how hemodynamic force regulates mouse yolk sac vascular remodeling *in vivo* has been largely aided by the application of imaging techniques and live embryo culture, which will be discussed later in this review (Jones et al., 2004, 2005; Larina et al., 2008, 2009a; Udan et al., 2013b). It is known that in the mouse, upon the initiation of the heartbeat at E8.25 (~3 somites) and the entry of blood cells into the circulation (~6 somites), hemodynamic force plays a critical role in the regulation of endothelial cell function and vascular remodeling, forming a low-resistance, highly organized yolk sac vasculature. The direct role of the onset of blood flow at this early stage of vascular development has been studied for several years and is still highly debated; some suggested that blood flow is necessary for the transport of oxygen to the growing embryonic tissue, while others suspected blood flow in the nascent vasculature is required for the transport of soluble nutrients and growth factors essential for proper endothelial cell function (Conway et al., 2003). However, it was also increasingly clear through *in vitro* experiments that shear stress, and therefore hemodynamic force, is a critical physical factor in the regulation of endothelial cell function. Shear stress (τ), the primary hemodynamic force required for vessel remodeling, is quantified by multiplying the apparent viscosity (μ_app_) of blood by the shear rate (γ), which is dependent on the velocity gradient of blood flow at the vessel wall and the vessel radius (Jones et al., 2004). Based on this definition, deficiencies in heart development, cardiac output, or hematopoiesis in the developing embryo affect the shear stress on a vessel wall, which can in turn significantly affect endothelial cell function and the ability of vessels to remodel during development.

Indeed, a number of mice with mutations causing deficiencies in early cardiac development and function are embryonic lethal due to impairments in vascular remodeling (Tanaka et al., 1999; Wakimoto et al., 2000; Koushik et al., 2001; Huang et al., 2003; May et al., 2004). For example, mutation of the gene atrial myosin light chain 2a *(mlc2a*), a structural component of the atrial myofibrillar apparatus, confers significantly reduced atrial contraction and reduced hemodynamic force (low, oscillatory flow), resulting in lethality by E10.5–11.5. Not only do embryos display cardiac morphogenesis abnormalities, but also a completely unremodeled yolk sac vasculature at E9.5 (Huang et al., 2003). Similarly, deletion of the Na^+^/Ca^2+^ exchanger (*Ncx1*) causes a complete lack of heartbeat and shows deficiencies in vascular remodeling (Wakimoto et al., 2000; Koushik et al., 2001). Furthermore, deletion of the homeobox transcription factor *Nkx2.5*, a regulator of dorsal mesoderm specification, results in impaired cardiac development after looping, defects in vascular remodeling in the yolk sac and embryo, and impairments in hematopoiesis. Interestingly, the authors demonstrated through chimera studies that vascular remodeling failure can be rescued if wild type *Nkx2.5* cardiomyocytes constitute greater than 85% of the heart (Tanaka et al., 1999), suggesting that cardiac function alone can play a significant role in the ability of vessels to remodel. Finally, embryos with mutations in the cardiomyocyte protein Titin, which acts as a spring during muscle contraction, have impaired blood flow, and endothelial cells of the yolk sac and embryo proper display defects in cell morphology, including irregular cell-cell contact and abnormal spatial distribution during vasculogenesis and angiogenesis (May et al., 2004). These data demonstrate the significance of cardiac output and its contribution to hemodynamic force during vascular development. In the absence of a strong heartbeat and the corresponding pulsatile, forward movement of blood flow within the capillary plexus, vessels within the yolk sac fail to remodel, and the embryo can no longer sustain growth resulting in lethality.

Shear stress imparted on blood vessels is dependent not only on blood velocity/cardiac output, but also on blood viscosity, which is correlated with the concentration of red blood cells within the circulation. The entry of blood cells into the embryonic circulation at ~6 somites is a necessary physiological cue for the induction of vascular remodeling. A number of gene mutations have been reported that result in the absence of hematopoietic development, where as a consequence, the vasculature fails to remodel (Robb et al., 1995; Matsumoto et al., 2006; He et al., 2008; Satyanarayana et al., 2010). For example, knockout of *Scl*, which encodes a bHLH transcription factor identified in T-cell leukemia, results in lethality by E9.5, due to significant developmental delay, edema, a complete absence of hematopoiesis, and an unremodeled yolk sac vasculature, suggesting that a significant reduction in blood development can impair yolk sac vascular remodeling (Robb et al., 1995). Similarly, deletion of the transcription factor Krüppel-like factor 6 (*Klf6*), which regulates cell proliferation, resulted in a drastic reduction in hematopoietic cell production and a poorly organized yolk sac vasculature. Upon the production of *Klf6*^−/−^ embryonic stem cells, it was determined that *Klf6* deletion resulted in a reduced capacity in hematopoietic lineage differentiation, while endothelial cell differentiation and expression remained unchanged (Matsumoto et al., 2006). More recently, deletion of the guanine nucleotide exchange factor *RapGEF2*, which activates Rap1, resulted in severe defects in yolk sac vascular remodeling. Although the vasculature failed to remodel, there were no changes in Flk1^+^ (endothelial) cells within the yolk sac, but a significant reduction in CD41^+^ (hematopoietic) cells, indicating the early blood and endothelial cell progenitors are still present, but maintenance of hematopoiesis no longer occurs (Satyanarayana et al., 2010). Also, conditional deletion of the proto-oncogene, *c-myc* in early blood and endothelial cell precursors resulted in a lack of early hematopoietic lineages, an impairment in definitive hematopoiesis, and deficient angiogenesis and yolk sac vascular remodeling. Furthermore, conditional *c-myc* deletion played no role in the establishment of the vascular plexus during angiogenesis, and had no effect on endothelial cell proliferation, migration or survival (He et al., 2008). These data indicate that the development of blood cells and their entry into the circulation at the onset of cardiac contraction is a physical trigger necessary for regulating dynamic endothelial cell function during vascular remodeling.

While there is strong evidence that cardiac output and the viscosity of blood are both necessary mechanical triggers for the activation of vascular remodeling through shear stress, until the past decade, there was no definitive answer to whether the onset of blood flow triggered yolk sac vascular remodeling through a purely mechanical mechanism in the mouse embryo. To answer this question *in vivo*, primitive erythroblasts were sequestered to the blood islands in cultured wild type embryos before the onset of blood flow through acrylamide polymerization, which reduced viscosity and shear stress within the circulation. In the absence of circulating red blood cells in cultured wild type embryos, the yolk sac capillary plexus did not remodel, and expression of endothelial nitric oxide synthase (eNOS), a known mechanosensitive protein, was significantly reduced. Furthermore, the remodeling defects and decreased eNOS expression levels observed in the cultured embryos with sequestered erythroblasts were rescued by the injection of a viscous material, hetastarch, into the circulation, which compensated for the lack of circulating blood cells (Lucitti et al., 2007). These studies show that the physical trigger of hemodynamic force and shear stress acting upon the endothelium is both necessary and sufficient to activate vascular remodeling in the mouse yolk sac.

## Live imaging of vascular remodeling and hemodynamics in the mouse embryo

Traditional developmental biology approaches to understanding how the vasculature changes over time during remodeling have taken advantage of immunohistochemistry and *in situ* hybridization to observe protein and gene expression within the endothelium at fixed time points. However, limiting observation to snapshots in time using these methods does not allow us to capture the complex, dynamic spatio-temporal changes of the remodeling vasculature. Since the advent of fluorescent proteins and genetic manipulation, the use of imaging techniques has been widely adapted for live analysis, such as cell migration, membrane dynamics, proliferation, and apoptosis. *In vivo* analysis of such cellular properties in the mouse during early development remains a significant challenge, due to the closed nature of mammalian *in utero* development. Within the past decade, mouse developmental biologists have begun to circumvent this limitation by combining embryo culture with imaging techniques to study vascular morphogenesis and hemodynamics in living embryos (Jones et al., 2004, 2005; Lucitti et al., 2007; Larina et al., 2008, 2009a; Garcia et al., 2011; Udan et al., 2013b).

**Figure 2:**
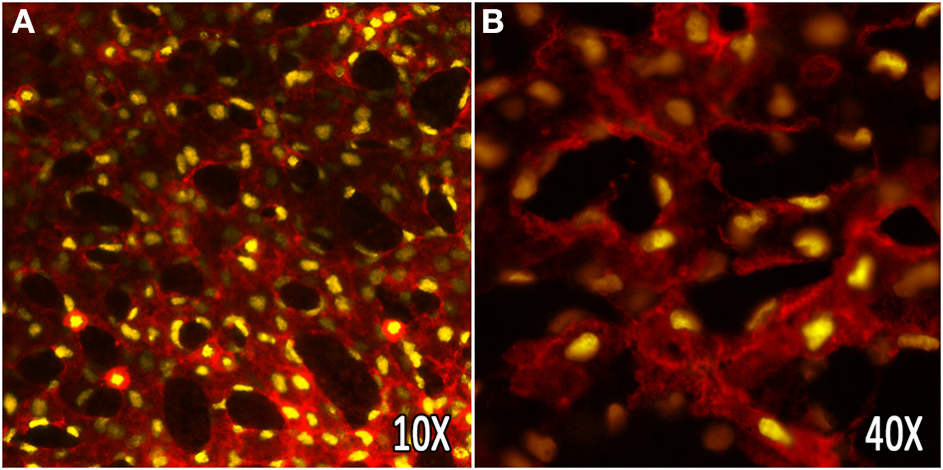
***In vivo* labeling of the yolk sac vasculature using vital fluorescent reporters**. The embryonic yolk sac vasculature of transgenic mice, labeling the membrane (Flk1-myr::mCherry), and nuclei (Flk1-H2B::YFP) of endothelial cells. **(A,B)** Vascular plexus of the embryonic yolk sac at E8.5 at 10× and 40× (oil) magnification, respectively.

Confocal microscopy has been used to image dynamic changes within the mouse yolk sac vasculature during remodeling. Transgenic mice expressing endothelial specific fluorescent reporters labeling the nucleus (Flk1-H2B::YFP) (Fraser et al., 2005) and the membrane (Flk1-myr::mCherry) (Larina et al., 2009b) have provided a straightforward imaging method to assess cell migration and vascular morphogenesis in cultured embryos (Figure 2). Embryonic imaging of these models in static culture on the imaging stage allows for visualization of the vasculature outline (labeled by the mCherry reporter) and tracking of all endothelial cells within the field of view, and has been used to study how endothelial cells respond to hemodynamic force during vascular remodeling (Udan et al., 2013b). Through time-lapse confocal microscopy, it has been shown that vessel diameter increases observed in the yolk sac vitelline artery during vascular remodeling are dependent on hemodynamic force, and are due to directed endothelial cell migration and vessel fusion events. Furthermore, when analyzing endothelial cell dynamics in blood flow-impaired mutant mice, the ability of endothelial cells to migrate and fuse to form larger vessels was lost. The dynamic endothelial behaviors observed using live imaging techniques have contributed to our knowledge of how endothelial cells respond to mechanical forces within the yolk sac during development, and highlighted the potential of live imaging for a more complete understanding of vascular morphogenesis in mouse embryos.

**Figure 3:**
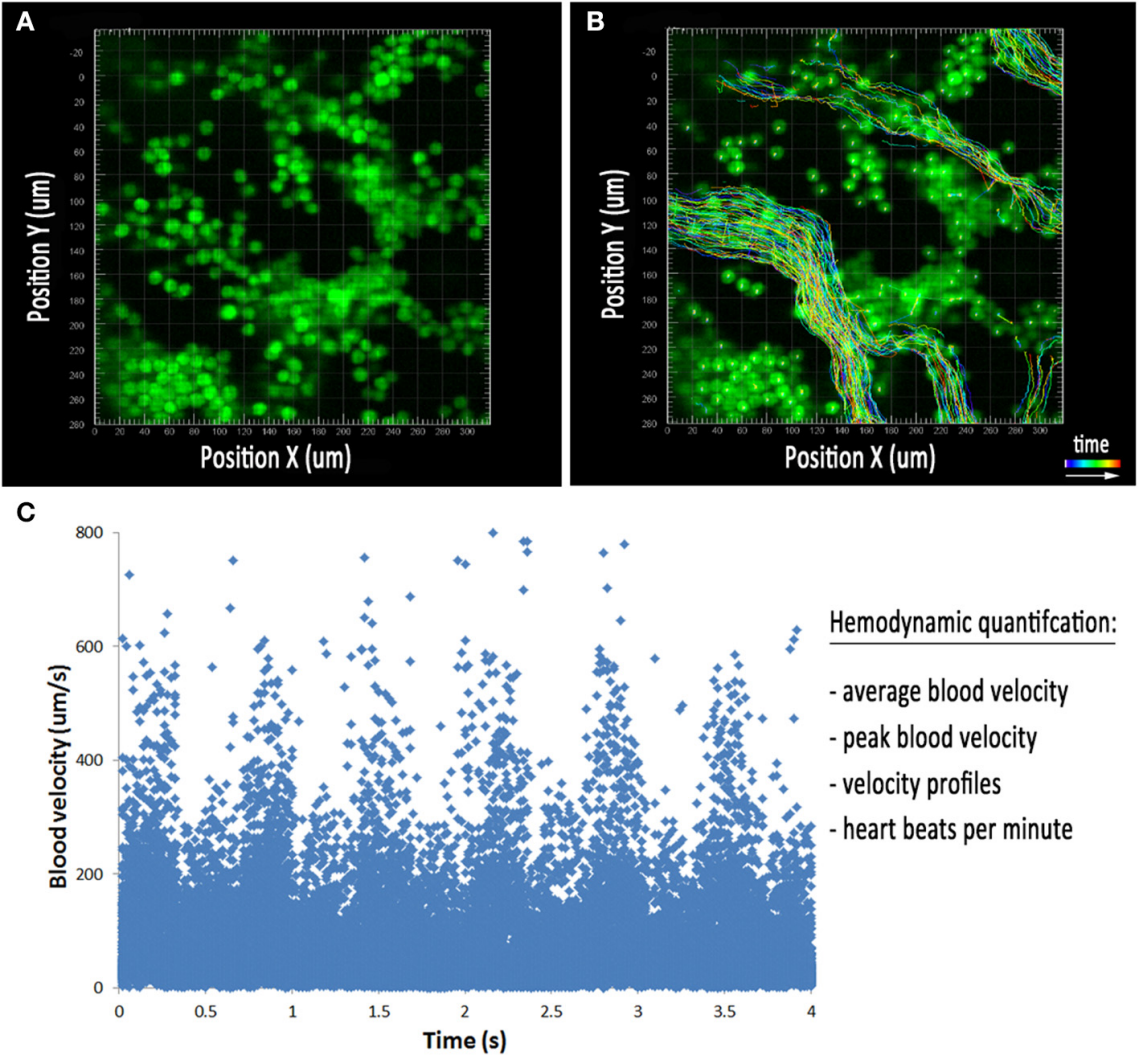
**Hemodynamic analysis in the embryonic yolk sac. (A)** Cultured embryos with erythrocytes labeled using the ε-globin-GFP transgene near the blood islands at E8.5. **(B)** Tracks of all cells detected using image processing software during the movie represented in **(A)**. The colored lines represent the position of the blood cells over the course of the time lapse. **(C)** Plot of individual cell velocities over the course of the time lapse. This allows for the quantification of average blood velocity, peak blood velocity, velocity profiles, and heart rate.

Time lapse microscopy of morphogenetic processes that occur over several hours can be easily performed using any number of conventional fluorescent microscopes. However, capturing the rapid, dynamic movement of blood flowing through a vessel using fluorescence microscopy can be challenging due to the high frame rate needed to resolve individual blood cells in time. Traditional confocal microscopes can be used for the acquisition and quantification of blood flow by repetitive, single line-scanning either parallel or perpendicular to the direction of flow (Dirnagl et al., 1992; Kleinfeld et al., 1998; Jones et al., 2004). However, this imaging technique provides a limited number of data points and rigorous validation. The development of line-scanning confocal microscopes allows for the rapid, real-time acquisition of blood flow *in vivo* (Dirnagl et al., 1992; Kleinfeld et al., 1998; Jones et al., 2004; Larina and Dickinson, 2012). Through the use of slits instead of conventional pinholes, line scanning confocal microscopes such as the Zeiss LSM 7 LIVE, which utilizes line illumination and a linear detector, have a frame rate of up to 120 frames per second. This rapid frame acquisition is ideal for imaging and tracking individual blood cells within a vessel over multiple frames for the quantification of blood velocity in the mouse yolk sac. Blood cells can be visualized in cultured embryos using the transgenic reporter mouse ε-globin-GFP, which brightly labels primitive erythroblasts during embryonic development (Figure 3A) (Dyer et al., 2001). Such fast frame rates allow cells to be easily distinguishable and traceable (Figure 3B), allowing a large number of cells per time lapse to be used for hemodynamic quantification, including peak blood velocity, average blood velocity, full blood velocity profiles, and heart rate (Figure 3C). This imaging technique provides a powerful tool for studying mouse mutants with impairments in vascular remodeling, helping to determine the direct cause of remodeling failure and distinguish between poor hemodynamics and endothelial cell dysfunction.

**Figure 4:**
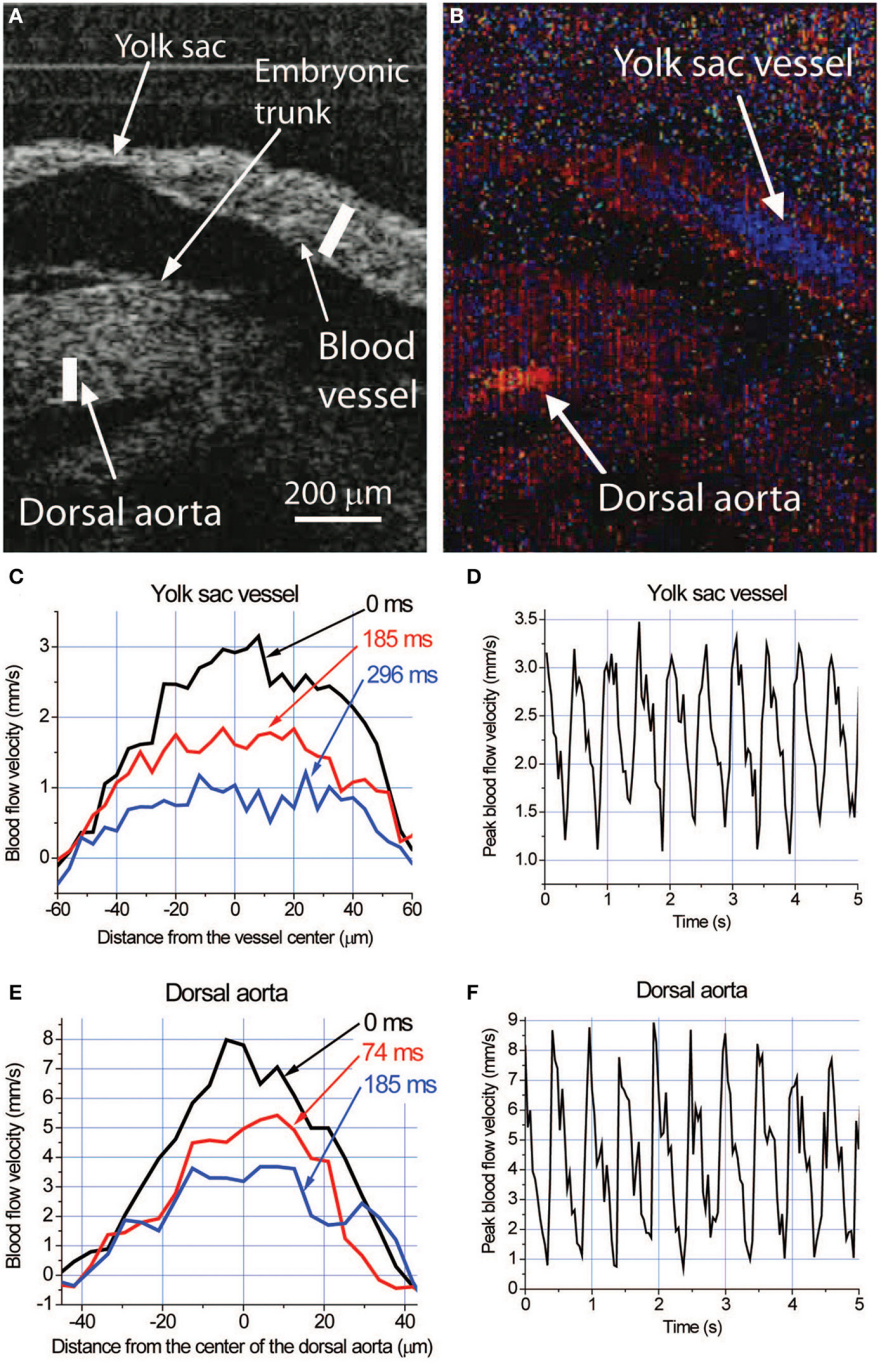
**Live hemodynamic analysis with Doppler OCT in cultured mouse embryos at E9.5. (A)** Structural image of an E9.5 embryo showing fragments of the yolk sac and embryonic trunk. **(B)** Corresponding color-coded Doppler OCT image showing strong signals produced by blood flow in the yolk sac vessel and dorsal aorta. Different colors indicate opposite directions of the flow in these structures. **(C,E)** Blood flow velocity profiles and **(D,F)** dynamics of the peak blood flow velocity in the yolk sac vessel and dorsal aorta, respectively, measured along the lines shown in **(A)** at different phases of the heartbeat cycle. Adapted from Larina et al. (2008).

Visualization of blood flow using confocal microscopy requires the use of fluorescent reporters or dyes through transgenic mice and/or embryo manipulation, which require time for breeding or is invasive. An alternative optical imaging technique, optical coherence tomography (OCT) takes advantage of interferometry to acquire images using natural optical tissue contrast, and does not require fluorescent proteins, dyes or contrast agents. OCT has the capability of imaging 2–3 mm into tissue, which enables deeper imaging within the cultured embryo, though with lower spatial resolution (~2–15 μm). We previously described the use of Doppler OCT to directly quantify blood velocity in the superficial vessels of the yolk sac, as well as deeper vessels within cultured embryos at E8.5 and E9.5 (Figure 4) (Chen et al., 1997). If the direction of the blood flow is known, blood flow at each pixel of an OCT image can be reconstructed. During vascular remodeling, blood velocity within the yolk sac and embryonic vessels is robust, allowing for a strong Doppler signal and an easy and reliable measurement of blood velocity in cultured embryos. Doppler OCT images are pseudocolored to indicate opposing blood flow directions in vessels (Figures 4A,B). Velocity measurements across both vessels result in a distinct parabolic velocity profile associated with blood flow, with the peak blood velocities at the center of the vessel, similar to that observed using confocal microscopy (Figures 4C,E). Analysis and quantification of the cardiac cycle can be performed by plotting the peak blood velocity observed within the vessel over time (Figures 4D,F). Hemodynamic data obtained by Doppler OCT in cultured mouse embryos has been validated as comparable to measurements made using fast-scanning confocal microscopy (Jones et al., 2004; Larina et al., 2008). Furthermore, while hemodynamic analysis of extraembryonic yolk sac vessels is relatively simple using confocal microscopy, the imaging depth limitations associated with fluorescence microscopy does not allow analysis of vessels deep within the embryo; this problem can be overcome using OCT. Also, analysis of OCT speckle variance within vessels in cultured embryos can be used to reconstruct the 3-D structure of the yolk sac and embryonic vasculature (Sudheendran et al., 2011; Mahmud et al., 2013). The development and use of OCT as an imaging tool for cultured embryos, specifically for cardiodynamic assessment, allows scientists to image deeper into the embryo without the need for fluorescent reporters, dyes or contrast agents, to answer fundamental questions about cardiovascular form and function in living mammalian embryos.

## Conclusion

Vascular remodeling, in which the primitive capillary plexus of the yolk sac is remodeled to form a branched hierarchical network of arteries, veins, and capillary beds, is a critical step in early cardiovascular development. As described in this review, impairments in this process can be due to defects in endothelial cell function that can inhibit the ability of the vasculature to respond to cues from signaling molecules, nutrients or physical forces. Secondarily, defects in hemodynamic force, either through altered cardiac output or hematocrit content, can also inhibit the ability of the vasculature to remodel. Although yolk sac vascular remodeling defects are fairly common, it is often the case that the direct cell type or mechanism for the impaired remodeling phenotype is not fully understood. Conditional knockout approaches directly targeting endothelial cells, cardiomyocytes, and/or hematopoietic cells, as well as imaging techniques, such as those described in this review, for *in vivo* analysis of hemodynamics and endothelial cell migration are all necessary when understanding the mechanism behind the complex morphological and molecular events that occur during vasculogenesis, angiogenesis, and vascular remodeling.

Basic research in vasculogenesis and angiogenesis has been supported by its clinical relevance, in which we can adapt what we learn to disease states such as cancer, atherosclerosis, and osteoporosis. Although a lot is known about the signal transduction pathways that regulate both vasculogenesis and angiogenesis, new genes are discovered every day, which can contribute to the regulation of endothelial cell function, vascular morphogenesis, and vascular homeostasis. Continuing studies that take advantage of animal disease models and genetic manipulations will be necessary to continue to elucidate the complexities of signal transduction and gene regulation during embryonic vascular development, to provide new gene targets and therapeutics for health intervention in the clinic.

### Conflict of interest statement

The authors declare that the research was conducted in the absence of any commercial or financial relationships that could be construed as a potential conflict of interest.
