# Corrigendum: Vascular development and hemodynamic force in the mouse yolk sac

**DOI:** 10.3389/fphys.2014.00492

**Published:** 2014-12-12

**Authors:** Monica D. Garcia, Irina V. Larina

**Affiliations:** Department of Molecular Physiology and Biophysics, Baylor College of MedicineHouston, TX, USA

**Keywords:** yolk sac, live imaging, vascular remodeling, mouse development, hemodynamics

The article “Vascular development and hemodynamic force in the mouse yolk sac” that is part of the research topic “Mechanotransduction and Development of Cardiovascular Form and Function” published 20 August 2014, is missing the following Acknowledgment Section:

## Acknowledgment

This work is supported by the National Institutes of Health (R01HL120140 and U54HG006348) as well as by the Optical Imaging and Vital Microscopy core at Baylor College of Medicine.

## Conflict of interest statement

The authors declare that the research was conducted in the absence of any commercial or financial relationships that could be construed as a potential conflict of interest.

